# Supplementary material for: Burden of tobacco-related cancers in urban, semi-urban and rural setting of Nepal: Findings from population-based cancer registries 2019
Source: PLoS One. 2024 May 28;19(5):e0300271. doi: 10.1371/journal.pone.0300271 (PMC11132438; doi:10.1371/journal.pone.0300271)
Supplement: Attachment — Submitted filename: review PONE-D-23-32604.docx [file pone.0300271.s002.docx]

PLOS ONE

Burden of tobacco-related cancers in urban, semi-urban and rural setting of Nepal: results from population-based cancer registries 2019

--Manuscript Draft--

| Manuscript Number: | PONE-D-23-32604 |
| --- | --- |
| Article Type: | Research Article |
| Full Title: | Burden of tobacco-related cancers in urban, semi-urban and rural setting of Nepal:  results from population-based cancer registries 2019 |
| Short Title: | Tobacco-related cancer burden in Nepal |
| Corresponding Author: | UMA KAFLE DAHAL, Master's Degree  Nepal Health Research Council  Kathmandu, Bagmati Province (3) NEPAL |
| Keywords: | Lung cancer, Nepal, Population-based Cancer Registry, Tobacco control, Tobaccorelated cancer. Rural and urban |
| Abstract: | Background Nepal is one of the high prevalent countries for tobacco use in Southeast Asia regions. Tobacco related cancer share the major burden since a decade, however, population-based estimates is still lacking. This study provides results from population-based cancer registries on tobacco-related cancer (TRCs) burden in Nepal. Methods The data were collected by population-based cancer registry conducted in nine districts by Nepal Health Research Council. The districts were categorized in urban, semi-urban and rural regions on the basis of geographical locations and facilities available. Analysis was done to identify tobacco-associated cancer incidence, mortality and patterns along with cumulative risk of having cancer before the age of 75 years. Results Tobacco-related cancer was 35.3% in men and 17.3% in women. Every one in 36 men and one in 65 women developed tobacco-related cancer in Nepal. Cancer of lung, mouth, esophagus and larynx were among the top five tobacco-related cancers in both males and females. The incidence of TRCs was higher in urban region with age adjusted rate 33.6 and 17.0 per 100,000 population for men and women respectively compared to semi-urban and rural regions. Tobacco-associated cancer mortality was significantly higher compared to incidence. Conclusion The prevalence of tobacco-related cancer remains high in Nepal despite of enforcement of tobacco control policy and strategies. Concerned authorities should focus towards monitoring of implemented tobacco control policy and strategies. |
| Order of Authors: | UMA KAFLE DAHAL, RN, BN, MSD |
|  | Meghnath Dhimal, PHD |
|  | Atul Budukh, PHD |
|  | Kopila Khadka, MA |
|  | Sudha Poudel, RN, MN |
|  | Gehanath Baral, MD |
|  | Pradip Gyanwali, MD |
|  | Anjani Kumar Jha, MD |
|  | Sandhya Chapagain, MD |
| Additional Information: |  |
| Question | Response |
| Financial Disclosure  Enter a financial disclosure statement that describes the sources of funding for the work included in this submission. Review | The author(s) received no specific funding for this work. |

| the [submission guidelines](http://journals.plos.org/plosone/s/submission-guidelines#loc-financial-disclosure-statement) for detailed requirements. View published research articles from [PLOS ONE](https://journals.plos.org/plosone/) for specific examples.  This statement is required for submission and will appear in the published article if the submission is accepted. Please make sure it is accurate. |  |
| --- | --- |
| Funded studies Enter a statement with the following details: - Initials of the authors who received each award - Grant numbers awarded to each author - The full name of each funder - URL of each funder website - Did the sponsors or funders play any role in the study design, data collection and analysis, decision to publish, or preparation of the manuscript? |  |
| Did you receive funding for this work? |  |
| Competing Interests  Use the instructions below to enter a competing interest statement for this submission. On behalf of all authors, disclose any [competing interests](http://journals.plos.org/plosone/s/competing-interests) that could be perceived to bias this work—acknowledging all financial support and any other relevant financial or nonfinancial competing interests.  This statement is required for submission and will appear in the published article if the submission is accepted. Please make sure it is accurate and that any funding sources listed in your Funding Information later in the submission form are also declared in your Financial Disclosure statement.  View published research articles from [PLOS ONE](http://journals.plos.org/plosone/) for specific examples. | The authors have declared that no competing interests exit. |

| NO authors have competing interests Enter: The authors have declared that no competing interests exist. Authors with competing interests Enter competing interest details beginning with this statement: I have read the journal's policy and the authors of this manuscript have the following competing interests: [insert competing interests here] |  |
| --- | --- |
| * typeset |  |
| Ethics Statement  Enter an ethics statement for this submission. This statement is required if the study involved:   - Human participants - Human specimens or tissue - Vertebrate animals or cephalopods - Vertebrate embryos or tissues - Field research   Write "N/A" if the submission does not require an ethics statement.  General guidance is provided below. Consult the [submission guidelines](http://journals.plos.org/plosone/s/submission-guidelines#loc-guidelines-for-specific-study-types) for detailed instructions. Make sure that all information entered here is included in the Methods section of the manuscript. | The study (Identifying incidence, trends, and patterns of cancer cases in Nepal:  National population-based cancer registry) was approved by ethical review board of Nepal health research council. The details of the study was registered in Ethical Review Board Registration number 436/2017. |

Format for specific study types

Human Subject Research (involving human

participants and/or tissue)

Give the name of the institutional review

board or ethics committee that approved the

study

•

Include the approval number and/or a

statement indicating approval of this

research

•

Indicate the form of consent obtained

(

written/oral) or the reason that consent was

not obtained (e.g. the data were analyzed

anonymously)

•

Animal Research (involving vertebrate

animals, embryos or tissues)

Provide the name of the Institutional Animal

Care and Use Committee (IACUC) or other

relevant ethics board that reviewed the

study protocol, and indicate whether they

approved this research or granted a formal

waiver of ethical approval

•

Include an approval number if one was

obtained

•

If the study involved

non-human primates

,

add

additional details

about animal welfare

and steps taken to ameliorate suffering

•

If anesthesia, euthanasia, or any kind of

animal sacrifice is part of the study, include

briefly which substances and/or methods

were applied

•

Field Research

Include the following details if this study

involves the collection of plant, animal, or

other materials from a natural setting:

Field permit number

•

Name of the institution or relevant body that

granted permission

•

Data Availability

Authors are required to make all data

underlying the findings described fully

available, without restriction, and from the

time of publication. PLOS allows rare

exceptions to address legal and ethical

concerns. See the

[PLOS Data Polic](http://journals.plos.org/plosone/s/data-availability)

[y](http://journals.plos.org/plosone/s/data-availability)

and

[FA](http://journals.plos.org/plosone/s/data-availability#loc-faqs-for-data-policy)

[Q](http://journals.plos.org/plosone/s/data-availability#loc-faqs-for-data-policy)

for detailed information.

Yes - all data are fully available without restriction

| A Data Availability Statement describing where the data can be found is required at submission. Your answers to this question constitute the Data Availability Statement and will be published in the article, if accepted. |  |
| --- | --- |
| Important: Stating ‘data available on request from the author’ is not sufficient. If your data are only available upon request, select ‘No’ for the first question and explain your exceptional situation in the text box. |  |
| Do the authors confirm that all data underlying the findings described in their manuscript are fully available without restriction? |  |
| Describe where the data may be found in full sentences. If you are copying our sample text, replace any instances of XXX with the appropriate details.   - If the data are held or will be held in a public repository, include URLs, accession numbers or DOIs. If this information will only be available after acceptance, indicate this by ticking the box below. For example: All XXX files   are available from the XXX database (accession number(s) XXX, XXX.).   - If the data are all contained within the manuscript and/or Supporting Information files, enter the following: All relevant data are within the manuscript and its Supporting Information files. - If neither of these applies but you are able to provide details of access elsewhere, with or without limitations, please do so. For example:   Data cannot be shared publicly because of [XXX]. Data are available from the XXX Institutional Data Access / Ethics Committee (contact via XXX) for researchers who meet the criteria for access to confidential data.  The data underlying the results presented in the study are available from (include the name of the third party | All relevant data are within the manuscript and its supporting information files. |

| and contact information or URL).  • This text is appropriate if the data are owned by a third party and authors do not have permission to share the data.  * typeset |  |
| --- | --- |
| Additional data availability information: |  |

Manuscript	[Click here to access/download;Manuscript;Tobacco-related](https://www.editorialmanager.com/pone/download.aspx?id=34455599&guid=5b9eb3c5-50f4-4d16-8c10-e82dcac93ddf&scheme=1)

[cancer burden in Nepal.docx](https://www.editorialmanager.com/pone/download.aspx?id=34455599&guid=5b9eb3c5-50f4-4d16-8c10-e82dcac93ddf&scheme=1)

Burden of tobacco-related cancers in urban, semi-urban and rural setting of Nepal: results from population-based cancer registries 2019

1 **Uma Kafle Dahal^1*^, Meghnath Dhimal^1^, Atul Budukh^4^, Kopila Khadka^1^, Sudha Poudel^1^ Gehanath**
2 **Baral^1^, Pradip Gyanwali^1^, Anjani Kumar Jha^3#b^, Sandhya Chapagain^2^**
3 1 Nepal Health Research Council, Ramshah Path, Kathmandu, Nepal
4 2 Department of Radiation Oncology, National Academy of Medical Sciences (NAMS), Bir Hospital, 5 Nepal
6 3 Department of Radiation Oncology, Kathmandu Cancer Center, Tathali, Bhaktapur, Nepal
7 4 Centre for Cancer Epidemiology (ACTREC), Tata Memorial Centre, Mumbai, Homi Bhabha National 8	Institute, Mumbai, India.

# 9 Abstract

10 **Background** Nepal is one of the high prevalent countries for tobacco use in Southeast Asia regions.
11 Tobacco related cancer share the major burden since a decade, however, population-based estimates is
12 still lacking. This study provides results from population-based cancer registries on tobacco-related
13 cancer (TRCs) burden in Nepal. **Methods** The data were collected by population-based cancer registry
14 conducted in nine districts by Nepal Health Research Council. The districts were categorized in urban,
15 semi-urban and rural regions on the basis of geographical locations and facilities available. Analysis was
16 done to identify tobacco-associated cancer incidence, mortality and patterns along with cumulative risk
17 of having cancer before the age of 75 years. **Results** Tobacco-related cancer was 35.3% in men and
18 17.3% in women. Every one in 36 men and one in 65 women developed tobacco-related cancer in
19 Nepal. Cancer of lung, mouth, esophagus and larynx were among the top five tobacco-related cancers in
20 both males and females. The incidence of TRCs was higher in urban region with age adjusted rate 33.6
21 and 17.0 per 100,000 population for men and women respectively compared to semi-urban and rural

regions. Tobacco-associated cancer mortality was significantly higher compared to incidence. **Conclusion** The prevalence of tobacco-related cancer remains high in Nepal despite of enforcement of tobacco control policy and strategies. Concerned authorities should focus towards monitoring of implemented tobacco control policy and strategies.

**Key words:** Lung cancer, Nepal, Population-based Cancer Registry, Tobacco control, Tobacco-related cancer. Rural and urban

# Introduction

Globally, the burden of cancer has been increased annually. According to GLOBOCAN, 19.3 million new cases and 10 million death of cancer were expected in the year 2020 (1) . Among total cancer reported in the world, 30-50% were preventable cancer (2). Tobacco control and lifestyle modification are the scientifically established preventable measures for cancer and also promotes cancer survival without complications (3). Based on the GLOBOCAN estimates for Nepal, more than twenty thousand (20805) new cancer cases and more than thirteen thousand (13629) deaths were reported in 2020 (4). As reported by latest results of Nepal burden of disease survey, malignant neoplasm shared 11% of total deaths among NCDs (5).

Tobacco use including smoking is one of major single preventable cause of death in the world (3) (6). WHO Framework Convention on Tobacco Control (WHO FCTC) is the first treaty negotiated in response to the globalization of tobacco epidemics. To reduce demand for tobacco use, WHO FCTC endorsed some measures regarding protection from exposure to tobacco smoke, tobacco contents regulation and its disclosure, packaging and labeling, education, communication, training and awareness, and tobacco advertising and sponsorship etc. Nepal had initiated tobacco control and prevention through endorsement of policies since 1990s, and the major intervention to control tobacco were taxation on international brands for sales and distribution of tobacco products and banning of tobacco advertisement on audio-visual media (7). Nepal signed the WHO FCTC on 3 December 2003 which was ratified from the house of representative on 7th November 2006. Nepal has enacted the

Tobacco Product Control Act 2011 which has incorporated key provision of WHO FCTC and

MPOWERs measures (8). MPOWER is an aggregated term for controlling tobacco product and it’s consumption where each words stands for Monitor tobacco use and Prevention policy, Protect people from tobacco smoke, Offer help to quit tobacco smoking, Warn about the dangers of tobacco, Enforce ban on tobacco advertising, promotion and sponsorship and Raise taxes on tobacco, respectively.

Moreover, tobacco demand reduction strategies were implemented with high priority through Nepal Health Sector Program (2010-2015) and the SDGs (2016-2030) has addressed to effectively implement the WHO FCTC to reduce prevalence of tobacco user in Nepal as well (8).

According to the results of global smoking epidemics, 32.6% and 6.5% adult men and women respectively smoke globally; the prevalence of smoking has dropped considerably in countries with higher socio-demographic countries. Some countries such as Nepal, the Netherlands, and Denmark showed substantially declined in smoking prevalence in women since 1990 (9). According to STEP wise approach to NCD risk factors surveillance in Nepal (2019), the prevalence of current tobacco user was 28.9% (48.3% men and 11.6% women) among them 17.1 % (28.0% men and 7.5% women) used tobacco smoking and 18.3% (33.3% men and 4.9% women) used smokeless tobacco product (10).

Tobacco contains more than 4000 chemicals including more than 60 known carcinogen in cigarette smoke and at least 16 in unburned tobacco (6). Tobacco use has been found significantly associated with the development of cancer in most part of the human body however, oral cavity, pharynx, larynx, lung, esophagus, pancreas, urinary bladder, and kidney cancer have been recognized as major tobacco-related cancers (TRCs) (11) (6). Cigarette smoking is responsible for 90% of lung cancer which is the leading cause of cancer related death globally, and cancer of the oral cavity (including lip and tongue) which is significantly associated with use of smokeless tobacco (6).

Results on burden of TRCs is one of important factors to guide policy makers to develop strategies on cancer prevention and control measures that are specific to tobacco use. The study aims to provide insightful results on incidence of TRCs based on population-based cancer registries (PBCR) data and its distribution throughout diverse geographical location of the country. The study will support national and provincial governments of Nepal to develop cancer prevention and control strategies including tobacco control.

# Method and Materials

Data was collected by the Population-based cancer registry (PBCR) Nepal which was operated by Nepal Health Research Council (NHRC) since 2018. Data were accessed on 14^th^ May 2023. Ethical approval was taken from ethical review board of Nepal health research council. The detail methodology of PBCR is described elsewhere in previously published report and articles (12) (13) (14). The PBCR Nepal has covered more than six million (21% of total estimated population of Nepal) population from different geographical regions (such as urban, semi-urban and rural) of the country. For the purpose of this study, all cancer sites which are recognized to be tobacco related cancer were computed along with their code of international classification of disease for oncology (ICD-O). Data on numbers, percentage, age adjusted rate (AAR), age specific crude rate for each TRCs sites for each gender and geographical regions were retrieved from published report. Cancer sites which were already known to be tobaccorelated were selected for this study. These are lip (C00), tongue (C01–C02), mouth (C03–C06), oropharynx (C10), hypo-pharynx (C12–C13), pharynx (C14), esophagus (C15), larynx (C32), lung (C33–C34), and urinary bladder (C67). Cumulative rate and cumulative risk for each TRCs was calculated using standard formula (5× ∑ [ASpR] ×100)/100000* and 100× (1 - exp [- cumulative rate/100]) respectively (15). The data related to cumulative risk of having tobacco-related cancer are presented in the clear and coherent way with the statement like one in number of individuals using 100/cumulative risk (%) method which is one of epidemiological indicators to express the burden of disease.

# Findings

## Tobacco related cancer incidence

The study found that out of 3295 new cases 851 were tobacco related cancers. The proportion of TRC among male and female was 35.3% and 17.3% respectively. The age adjusted rate of TRC for males and females was 23.2 and 12.7 (per 100000 people) respectively. The overall cumulative lifetime risk (074years) of tobacco-related cancers in Nepal revealed that 1 in 36 for males and 1 in 65 for females. The highest incidence of TRC for men (AAR 33.6 per 100,000) and women (AAR 17.0 per 100,000) was found in urban region as compared to semi-urban and rural region which is shown in Table 1.

**Table ^[1]^:** Incidence of tobacco-related cancer versus all cancer among men and women in different regions of Nepal based on the locations of population-based cancer registry

|  | **Men** | |  | | **Women** | | |  | | **Men** | | **Women** | | | |
| --- | --- | --- | --- | --- | --- | --- | --- | --- | --- | --- | --- | --- | --- | --- | --- |
| **Region of registry** | TRC (%TRCs of all cancer) | | All cancer | | TRC (%TRCs of All cancer all cancer) | | | TRC | | All cancer | | TRC | | All cancer | |
| **Urban** | 33.6 (36.2) | | 86.7 | | 17.0 (17.3) | | 90.8 | 24 | | 10 | | 47 | | 10 | |
| **Semi-Urban** | 16.5 (34.4) | | 47.8 | | 8.7 (16.2) | | 49 | 50 | | 21 | | 95 | | 18 | |
| **Rural** | | 16.6 (30.2) | | 52.5 | | 15.3 (31.1) | 47.3 | | 50 | | 17 | | 67 | | 21 |
| **All regions** | | 23.4 (35.3) | | 63.5 | | 12.7 (17.3) | 68.1 | | 36 | | 14 | | 65 | | 13 |

The highest incidence among male was observed for the lung cancer with AAR 9.8 per 100,000 population followed by mouth (AAR 4.1), bladder (AAR 2.6), larynx (AAR 2.0) and esophagus (AAR,

1.7) respectively. Similarly, in the females, the leading TRCs was found among the lung cancers with

AAR 7.7 followed by tongue (AAR 1.2), esophagus (AAR 1.0), mouth (AAR 0.9), and larynx (AAR

0.7) respectively.

## Tobacco-related cancer deaths

Out of 1427 registered deaths cases 32.3% (male 41% and female 22.5%) were TRCs. The age adjusted mortality of TRCs per 100,000 persons was 13.1 for male and 6.3 for female. Lung cancer was the major cause of death which comprised 24.1% (AAR 7.9 per 100,000) of total death among male followed by mouth 4.7% (AAR 1.5) and esophagus 3.2% (AAR 1.0), larynx 2.4% (AAR 0.8) and bladder 1.8% (AAR 0.6). Similarly, the lung cancer contributed the highest proportion (17.1%) of death among females with age adjusted rate 4.9 per 100,000 populations. Followed by lung cancer, the common cause of death among TRCs was esophagus 1.3% (AAR 0.3). Mouth and tongue cancer comprised equal proportion (1%, AAR 0.3) of death cases, and bladder cancer covered 0.9% of total death with AAR 0.3 per 100,000. , Lung cancer was the commonest cause of death identified in each geographical with higher proportion in rural (male 34.8% and female 17%) and urban region (male 33.1% female 22.6%) in comparison to semi-urban regions. The mouth cancer which is an important contributor of smokeless tobacco use was higher (male 8.7% and female 2.9%) in semi-urban regions of the country.

## Cumulative risk (lifetime) of having cancer (0-74 years)

The risk of having lung cancer was the highest in both sex with 1 in 79 men and 1 in 106 women followed by mouth cancer (1 in 216) in males and tongue cancer in females with the risk of 1 in 613 women. The development of cancer of oropharynx and pharynx were identified as the lowest risk in Nepal with 1 in 3,425 men and 1 in 17,857 women respectively.

The Kathmandu valley (urban region) followed by the Rukum (rural hill) was at the highest risk for developing lung cancer with 1 in every 45 and 1 in 61 men and 1 in 70 and 1 in 68 women respectively. Similarly, the SSDM (semi-urban) regions have greatest risk for developing mouth cancer followed by lung in males with 1 in 141 and 1in 174 men respectively, and in females the risk was vice-versa with 1 in 199 and 1 in 612 women for lung and mouth cancer respectively. The number of TRC cases, proportion of TRC with all cancer, age adjusted rate (AAR), and cumulative risk percentage for each TRC sites for males and female of each PBCR locations is presented in table 2 and 3 respectively.

**Table 2:** Incidence of tobacco-related cancers in different geographical regions of Nepal based on locations of population-based cancer registry, Males

| PBCR region |  | Lip (C00) | Tongue  (C01-  02) | Mouth  (C03-  06) | Oropharynx (C10) | Hypopharynx (C12-13) | Pharynx (C14) | Esophagus (C16) | Larynx (C32) | Lungs  (C33-  34) | Bladder (C67) |
| --- | --- | --- | --- | --- | --- | --- | --- | --- | --- | --- | --- |
| Urban | N  %  AAR | 3  0.3  0.3 | 20  2.1  1.9 | 26  2.8  2.4 | 7  0.7  0.6 | 12  1.3  1.2 | 2  0.2  0.2 | 27  2.9  2.6 | 34  3.6  3.5 | 165  17.6  16.7 | 43  4.6  4.2 |
|  | Cum  Risk  % (*) | 0.0277 (3610) | 0.2164 (462) | 0.2454 (407) | 0.0694 (1441) | 0.1633 (612) | 0.0575 (1739) | 0.3197 (313) | 0.4698 (213) | 2.2402  (45) | 0.4875 (205) |
| Semi  -  urban | N  %  AAR | 7  1.2  0.5 | 15  2.6  1.2 | 75  13  6.2 | -  -  - | 5  0.9  0.4 | 4  0.7  0.3 | 13  2.2  1.2 | 11  1.9  1.0 | 54  9.3  4.4 | 15  2.6  1.3 |
|  | Cum  Risk  % (*) | 0.0879 (1138) | 0.1321 (757) | 0.7074 (141) | - | 0.0607 (1647) | 0.036  (2778) | 0.1092 (916) | 0.114 (877) | 0.5746 (174) | 0.1314 (761) |
| Ru  ral | N | - | - | - | - | - | - | - | 2 | 8 | 3 |

|  | % | - | - | - | - | - | - | - | 4.7 | 18.6 | 7 |
| --- | --- | --- | --- | --- | --- | --- | --- | --- | --- | --- | --- |
|  | AAR | - | - | - | - | - | - | - | 2.4 | 9.7 | 4.4 |
|  | Cum  Risk  % (*) | - | - | - | - | - | - | - | 0.1308 (765) | 1.6475  (61) | 0.254 (394) |
| All regions | N  %  AAR | 10  0.6  0.4 | 35  2.2  1.4 | 101  6.5  4.1 | 7  0.4  0.3 | 17  1.1  0.7 | 6  0.4  0.2 | 40  2.6  1.7 | 47  3.0  2.0 | 227  14.6  9.8 | 61  3.9  2.6 |
|  | Cum  Risk  % (*) | 0.0614 (1629) | 0.15 36 (651) | 0.4639 (216) | 0.0292 (3425) | 0.1021 (979) | 0.0400 (2500) | 0.1843 (543) | 0.2523 (396) | 1.2647  (79) | 0.2719 (368) |

137

138	Note: N-Number of incidence cases, %- relative proportion of TRCs among all cases, AAR- age adjusted rate per 139	100,000 population and (*) denotes one in number of person (0-74 Years) likely to develop cancer in lifetime.

140

**Table 3**: Incidence of tobacco-related cancers in different geographical regions of Nepal based on locations of population-based cancer registry, Females

| PBCR regions | | Lip (C00) | Tongue  (C01-  02) | Mouth  (C03-  06) | Oropharynx (C10) | Hypopharynx (C12-13) | Pharynx (C14) | Esophagus (C16) | Larynx (C32) | Lungs  (C33-  34) | Bladder (C67) |
| --- | --- | --- | --- | --- | --- | --- | --- | --- | --- | --- | --- |
| Urban | N  %  AAR | 3  0.3  0.2 | 17  1.6  1.7 | 7  0.6  0.6 | 1  0.1  0.1 | 3  0.3  0.2 | -  -  - | 19  1.8  1.6 | 7  0.6  0.8 | 122  11.3  11 | 8  0.7  0.7 |
|  | Cum  Risk %  (*) | 0.0313 (3195) | 0.2387 (419) | 0.497  (2012) | 0.0051  (19608) | 0.0224 (4464) | - | 0.1978 (506) | 0.1113 (898) | 1.4199  (70) | 0.0820 (1220) |
| N  %  -  urban    AAR | | -  -  - | 11  1.8  1.0 | 14  2.3  1.2 | 3  0.5  0.3 | 1  0.2  0.1 | 1  0.2  0.1 | 5  0.8  0.4 | 9  1.5  0.8 | 49  8.0  4.4 | 6  1.0  0.5 |
| Semi | Cum  Risk %  (*) | - | 0.1185 (844) | 0.1633 (612) | 0.0435 (2299) | 0.0151 (6623) | 0.0100  (10000) | 0.0681 (1468) | 0.0723 (1383) | 0.5037 (199) | 0.0623 (1605) |
| Rural | N  %  AAR | -  -  - | 1  2.22  0.6 | -  -  - | -  -  - | -  -  - | 1  2.22  1.3 | -  -  - | 1  2.22  1.3 | 10  22.2  10.9 | 1  2.22  1.3 |
|  | Cum  Risk %  (*) | - | 0.0316 (1365) | - | - | - | - | - | - | 1.475  (68) | - |
| N  %  regions    AAR | | 3  0.2  0.1 | 29  1.7  1.2 | 21  1.2  0.9 | 4  0.2  0.2 | 4  0.2  0.2 | 2  0.1  0.1 | 24  1.4  1.0 | 17  1.0  0.7 | 181  10.4  7.7 | 15  0.9  0.6 |
| All | Cum  Risk %  (*) | 0.0150 (6667) | 0.1631 (613) | 0.1024 (977) | 0.0250 (4000) | 0.0180 (5556) | 0.0056  (17857) | 0.1220 (820) | 0.0854 (1171) | 0.9396 (106) | 0.0684 (1462) |

Note: N-Number of incidence cases, %- relative proportion of TRCs among all cases, AAR- age adjusted rate per 100,000 population and (*) denotes one in number of person (0-74 Years) likely to develop cancer in lifetime.

# Discussion

The study finding showed that more than one third (35.3%) among men and less than one fifth (17.3%) of all cancer among women were tobacco-related. There was slight reduction in the overall proportion of

TRCs in 2019 compared to 2018 (men 36.3% and women 14.7%) in Nepal this may be due to underreporting of cancer cases; however, ongoing surveillance will provide further stability on cancer burden. The scientific community still lacks the supporting results on tobacco-related cancer in Nepal except one hospital-based study which was conducted in one hospital in western Nepal that showed higher proportion (male 48% and female 28%) of tobacco-associated cancer in both gender (16). The proportion of TRCs in different regions of India range from 11–25% for men and 3–18% for women, and the overall share of TRCs in India was 45% and 20% for male and female respectively which was higher compared to Nepal (11). The PBCR of Varanasi in India reports the highest burden of TRCs in India. The TRC burden in Nepal for males is low as compared to Varanasi and other Indian registries such as Sangrur, Chandigarh, Delhi, Barshi rural, Mumbai and Chennai as well as other registries in United Kingdom, United States and Curitiba, Brazil. However, the burden of TRC for females in Nepal is higher as compared to Varanasi and above-mentioned registries (17). The Muzaffarpur district of

India shared southern border of Madhesh province of Nepal where Siraha, Spatari, Dhanusha and Mohattari (SSDM) PBCR had conducted. Tobacco-related cancer in Muzaffarpur district among men was 36.5% which was almost similar with the TRCs among men in semi-urban region (SSDM registry) of Nepal. However, the proportion among women (8.8%) was almost 50% lower compared to women in in Nepal (18).

Approximately one third (32.3%) of all cancer deaths (male 41% and female 22.5%) was identified to be associated with tobacco in Nepal. The share of death associated to be TRCs among male in Nepal was almost comparable with the results in India where 42.0% male and 18.3% female death was due to tobacco use (19). The proportion of smoking related death in china showed 28.87% among males and 13.37% among females (15) which was considerably lower compared to Nepal.

Every one in 80 men and 107 women are at risk of getting lung cancer before age of 75 in Nepal, however, the risk for lung cancer in India (1 in 67 person) was less compared to Nepal (11). Lung cancer is the commonest cause of tobacco associated cancer in most developing countries including China, and also in the developed country like United States (21) (22). Lung cancer in Nepal is common as well as the most fetal cancer in Nepal and covered major share of all incidence and death cases. It has been remained the leading cancer and commonest cause of death since long in Nepal (23) (24) (25). The proportion of lung cancer among female of rural region was increased significantly from 9.3% in 2018 to 22.2% in 2019 this may be due to improvement in death registration (26) (12). The most important known cause of lung cancer is tobacco smoking which contain majority of human carcinogens (6) and long term exposure to pollution (27). Overall 17.1% people smoke tobacco in Nepal (10). Increase in incidence of lung cancer may be associated with the long term daily exposure to smoke from unprocessed biomass fuels produced during burning of wood, dung, and crop residues. Results showed that people in developing countries mostly in rural household especially women are commonly exposed to high levels of indoor pollution for 3–7 hour everyday over many years for their daily work (i.e. Cooking) (28).

Unburned tobacco product such as oral snuff, chewing tobacco (Gutkha, Khaini, Surti, Areca nuts and Betel leaf etc.) contain relatively low level of carcinogens compared to smoked tobacco, however, they are known to be responsible for causing cancer of oral cavity (6) (29). Overall 18.3% of current tobacco user in Nepal use smokeless tobacco product, and two third (71.4%) of them use surti (dried tobacco leaves) or khaini (lime missed tobacco). The male to female ratio of smokeless tobacco user in Nepal was 6.7 (10). Consequently, cancer of oral cavity (including lip, tongue, mouth and pharynx) in Nepal was found significantly higher in males (11.2%) compared to females (3.6%) which was comparable with the results found in Sri Lanka (30) as well as Europe (31). Every one in 216 men and 1 in 977 women is at risk for mouth cancer in Nepal which was likely to be similar with a the finding in India that shows the overall risk of mouth cancer was 1 in 250 in the southern and central regions of the country (11).

The risk for esophageal cancer in Nepal (1 in 543 men and 1 in 820 women) is significantly lower compared to 1 in 27 people in India especially in Northeastern regions (11), however, results shows high prevalence of chewing areca nuts (plain Supari) in Nepal (32). Areca nuts chewing was found associated with oral and esophageal cancer (33).

Nepal has been implementing operational policies and action plan since more than a decade through Tobacco Product Control Act 2011 and WHO FCTC MPOWER measures which includes making all public places smoke-free; providing large, legible warning labels; increasing tax on tobacco products as well as banning advertisement on national television, radio, and print media etc.; despite operation of such tobacco control programs, there was no subsequent decreased in the proportion of tobacco related cancer in Nepal. Moreover, the proportion of current smokers who tried to quitting tobacco use was significantly decreased from 26.1% in 2013 to 19.4% in 2019. In India, 55.4% current tobacco smokers and 49.6% smokeless tobacco user were thinking to quit tobacco which was almost two fold higher in compared to Nepal. The mean age of initiating tobacco smoking in Nepal remains same since 2013 (34).

The result suggests to focus on monitoring of tobacco control strategies and its effectiveness along with the introduction of new effective educational and awareness modalities such as behavioral counseling and active follow up which can help the quitters to quit tobacco successfully. Group or individual psychological support through behavioral counseling using the steps 5 A’S (Ask about tobacco use, Assess to quit, Assess commitment and barrier to change, Assist user committed to change and Arrange follow-up to monitor progress) to those who were willing to quit is cost effective approach to reduce habit-associated ill health. Furthermore, educational advocacy and behavioral counseling using 5 R’s model (Relevance, Risk, Rewards, Roadblocks and Repetition) can help tobacco user to quit tobacco successfully although they were not willing to quit tobacco (35). Cancer awareness through school education with structured session planning including early signs, symptoms, risks factors, preventive measures (such as stopping smoking and tobacco use), and treatment modalities helps to promote disease awareness and support early detection (36). It is recommended that Tobacco Quitline Services for tobacco control should be started in Nepal as these services are beneficial in India (37) (38).

Last but not the least, authors want to explore some limitation of the present study that the study provide tobacco-related cancer statistics in all geographical regions of the country; however, it does not cover entire population of the regions because of limited population coverage by PBCR. Moreover, population–based cancer registry does not registered information on tobacco use and other risk factors. Thus, the findings was analyzed based on the site of cancer that are known to be associated with tobacco use, however, it does not estimate the proportion of cancer directly caused by tobacco use.

# Conclusion

The prevalence of tobacco associated cancer remains high in Nepal, in all geographical regions of the country. Government of Nepal should give priority to strong implementation of tobacco control strategies and perform periodic monitoring of its execution. There should be a tobacco quit helpline that provide behavioral counseling to individuals who try to quit. Population-based cancer registries should be government priority and expanded to all provinces to monitor the burden of cancer in the country.

# Acknowledgement

We would especially want to thank the Secretary of the Ministry of Health and Population (MoHP),

Government of Nepal, for their invaluable support and encouragement in helping Nepal to initiate the Population-Based Cancer Registry (PBCR). We really appreciate the continuous technical assistance for data quality monitoring and capacity building provided by the International Agency for Research on

Cancer (IARC) Regional Hub at Tata Memorial Center, Mumbai, India and the WHO Country Office Nepal. Our deepest appreciation goes to the Health Tax Fund under ministry of health and population for their continuous support to sustain the registry in Nepal. Thank you to everyone who helped establish the PBCR in the urban, semi-urban and rural setting of the country including the local government, cancer treatment and diagnostic facilities, hospices social security and nursing division under the department of health services. Finally, we would like to thank all registry staffs for their dedication and effort to run PBCR in Nepal.

# References

1. Sung H, Ferlay J, Siegel RL, Laversanne M, Soerjomataram I, Jemal A, et al. Global Cancer

Statistics 2020: GLOBOCAN Estimates of Incidence and Mortality Worldwide for 36 Cancers in 185 Countries. CA Cancer J Clin. 2021 May 1;71(3):209–49. https://doi.org/10.3322/caac.21660

2. International Agency for Research on Cancer. GLOBOCAN 2020, New Global Cancer Data | UICC. https://www.uicc.org/news/globocan-2020-new-global-cancer-data [Accessed 2022 Nov

23]

3. Balogh EP, Dresler C, Fleury ME, Gritz ER, Kean TJ, Myers ML, et al. Reducing TobaccoRelated Cancer Incidence and Mortality: Summary of an Institute of Medicine Workshop. The

Oncologist. 2014 Jan;19 (1):21. https://doi.org/10.1634%2Ftheoncologist.2013-0230

4. International Agency for Research on Cancer. GLOBOCAN Nepal 2020, The Global Cancer Observatory. Available from: https://gco.iarc.fr/today/data/factsheets/populations/524-nepal-factsheets.pdf
5. Nepal Health Research Council. Nepal Burden of Disease 2019, A Country Report based on the

Global Burden of Disease 2019 Study. https://nhrc.gov.np/wp-content/uploads/2022/02/BoD-

Report-Book-includ-Cover-mail-6_compressed.pdf [Accessed 2022 Nov 17]

6. Hecht SS. Tobacco carcinogens, their biomarkers and tobacco-induced cancer. Nat Rev Cancer. 2003 Dec;3(10):733–44. https://doi.org/10.1038/nrc1190
7. Khanal GN, Khatri RB. Burden, prevention and control of tobacco consumption in Nepal: a narrative review of existing evidence. Int Health. 2021 Feb 24;13 (2):110–21.

https://doi.org/10.1093/inthealth/ihaa055

8. Nepal Government. The Ministry of Health, FCTC 2030 Strategy Nepal 2018 Available from: https://www.nepalindata.com/media/resources/items/20/bNational-Tobacco-Control-StrategicPlan-2013-2016-Final-11-March-2013.pdf [Accessed 2023 Jun 2].
9. Xiaochen Dai, Emmanuela Gakidou, Alan D Lopez. Evolution of the global smoking epidemic over the past half century: strengthening the evidence base for policy action. 2022.

https://tobaccocontrol.bmj.com/content/tobaccocontrol/31/2/129.full.pdf [Accessed 2023 Apr 6]

10. Non-Communicable Disease Risk Factors: Steps Survey Nepal 2019. Available from:

https://www.who.int/docs/default-source/nepal-documents/ncds/ncd-steps-survey-2019compressed.pdf?sfvrsn=807bc4c6_2 [Accessed 2022 Mar 23]

11. Asthana S, Patil RS, Labani S. Tobacco-related cancers in India: A review of incidence reported from population-based cancer registries. Indian J Med Paediatr Oncol. 2016 Jul;37 (03):152–7. https://doi.org/10.4103/0971-5851.190357 [Accessed 2023 Jan 4 ]
12. Nepal Health Research Council. Cancer incidence and mortality rate in selected district of Nepal in 2019. https://nhrc.gov.np/wp-content/uploads/2022/08/Population-Based-Cancer-Registry-

Report-2019-1.pdf

13. Subedi R, Dhimal M, Budukh A, Chapagain S, Gyanwali P, Gyawali B, et al. Epidemiologic

Pattern of Cancer in Kathmandu Valley, Nepal: Findings of Population-Based Cancer Registry, 2018. JCO Glob Oncol. 2021 Dec;(7):443–52. https://doi.org/10.1200/GO.20.00574

14. Subedi R, Budukh A, Chapagain S, Gyanwali P, Gyawali B, Khadka K, et al. Differences in cancer incidence and pattern between urban and rural Nepal: one-year experience from two

population-based	cancer	registries.	ecancermedicalscience	2021

https://www.ncbi.nlm.nih.gov/pmc/articles/PMC8183641/ [Accessed 2023 Jul 20]

15. Jensen OM., Parkin D.M., Maclennan R., Muir C.S., Kkeet R.G. Cancer Registration: Principles and Methods. IARC; 1991. 295 p.
16. Binu V, Chandrashekhar T, Subba S, Jacob S, Kakria A, Menezes RG. Cancer Pattern in

Western	Nepal:	A	Hospital	Based	Retrospective	Study.

https://journal.waocp.org/?sid=Entrez:PubMed&id=pmid:17696728&key=2007.8.2.183

17. Budukh AM, Pradhan S, Singh VB, Khanna D, Bagal SS, Chakravarti PS, et al. Cancer pattern in Varanasi district from Uttar Pradesh state of India, a foundation for cancer control based on

the first report of the population-based cancer registry. Indian J Cancer. 2023 Feb 27; DOI: 10.4103/ijc.IJC_44_21

18. Budukh A, Bagal S, Pandey N, Gupta D, Singh R, Chaturvedi P, Dikshit R, and Badwe R. Cancer Incidence and Mortality in Muzaffarpur, Bihar state, India: 2018. Tata Memorial Centre

(TMC),	Mumbai,	India

(2023).https://tmc.gov.in/tmh/pdf/Reports/Muzaffarpur%20PBCR_2018.pdf

19. Dikshit R, Gupta PC, Ramasundarahettige C, Gajalakshmi V, Aleksandrowicz L, Badwe R, et al.

Cancer mortality in India: a nationally representative survey. The Lancet. 2012 May 12;379(9828):1807–16. https://doi.org/10.1016/S0140-6736(12)60358-4

20. Li W, Xue X, Li D, Zhang Y, Shen W, Pan Y, Wang D, Zheng W, Jiang G. Attributable fraction of tobacco smoking on selected cancer deaths in the past decade using mortality case-control

study	in	Tianjin,	China.	Tob	Induc	Dis.	2022	Sep	5;20:75.2022

https://doi.org/10.18332/tid/152203 [Accessed 2023 Jun 18]

21. Wang JB, Jiang Y, Wei WQ, Yang GH, Qiao YL, Boffetta P. Estimation of cancer incidence and mortality attributable to smoking in China. Cancer Causes Control. 2010 Jun;21(6):959–65. https://doi.org/10.1007/s10552-010-9523-8
22. Gallaway MS, Henley SJ, Steele CB, Momin B, Thomas CC, Jamal A, et al. Surveillance for Cancers Associated with Tobacco Use — United States, 2010–2014. MMWR Surveill Summ. 2018 Nov 2;67(12):1–42. https://doi.org/10.15585/mmwr.ss6712a1
23. Nepal Health Research Council. Cancer Incidence and Mortality in Kathmandu Valley in 2018.

Available from: https://nhrc.gov.np/wp-content/uploads/2020/08/KTM-Valley-Report-final.pdf

24. Shrestha G, Thakur RK, Singh R, Mulmi R, Shrestha A, Pradhan PMS. Cancer burden in Nepal,

1990–2017: An analysis of the Global Burden of Disease study. PLOS ONE. 2021 Aug 3;16(8):e0255499. https://doi.org/10.1371/journal.pone.0255499

25. Krishna Kanta Poudel, , Zhibi Huang, , Prakash Raj Neupane. Trend of Cancer Incidence in Nepal from 2003 to 2012. Asian Pac J Cancer Prev 17 4 2171-2175. 2016;

https://doi.org/10.22034/APJCP.2017.18.1.165

26. Nepal health research council. Cancer incidence and mortality in Rukum district in 2018. Retrieved from: https://nhrc.gov.np/wp-content/uploads/2020/08/Rukum_Report_final-2.pdf
27. Cohen AJ. Air pollution and lung cancer: what more do we need to know? Thorax. 2003 Dec 1;58(12):1010–2. https://doi.org/10.1136/thorax.58.12.1010
28. Ranabhat CL, Kim CB, Kim CS, Jha N, Deepak KC, Connel FA. Consequence of Indoor Air

Pollution in Rural Area of Nepal: A Simplified Measurement Approach. Front Public Health. 2015 [cited 2022 Nov 28];3. https://doi.org/10.3389/fpubh.2015.00005

29. Warnakulasuriya S, Chen THH. Areca Nut and Oral Cancer: Evidence from Studies Conducted in Humans. J Dent Res. 2022 Sep;101(10):1139. https://doi.org/10.1177/00220345221092751
30. Amarasinghe AAHK, Usgodaarachchi US, Johnson NW, Warnakulasuriya S. High Prevalence of

Lifestyle Factors Attributable for Oral Cancer, and of Oral Potentially Malignant Disorders in

Rural	Sri	Lanka.	Asian	Pac	J	Cancer	Prev	APJCP.	2018;19(9):2485–92.

https://doi.org/10.22034/APJCP.2018.19.9.2485

31. Convergence of decreasing male and increasing female incidence rates in major tobacco-related

cancers	in	Europe	in	1988–2010.	Eur	J	Cancer.	2015	Jun	1;51(9):1144–63.

https://doi.org/10.1016/j.ejca.2013.10.014

32. Wazir SS, Arora P, Kapoor S, Jayam R, Sharma S, Rastogi T. Prevalence of areca nut chewing habit among high school children of Parsa district of Nepal. J Oral Biol Craniofacial Res. 2017 Dec;7(3):161. https://doi.org/10.1016/j.jobcr.2016.10.008
33. Li J, Xu J, Zheng Y, Gao Y, He S, Li H, et al. Esophageal cancer: Epidemiology, risk factors and screening. Chin J Cancer Res. 2021;33(5):535–47. https://doi.org/10.21147/j.issn.1000-

9604.2021.05.01

34. Global-Adult-Tobacco-Survey-Second-Round-India-2016-2017.pdf [Internet]. [cited 2023 Sep 15]. Available from: https://ntcp.mohfw.gov.in/assets/document/surveys-reportspublications/Global-Adult-Tobacco-Survey-Second-Round-India-2016-2017.pdf
35. D.C M, Shekar V, Reddy R.C J. Effect of Behavioral counseling in Tobacco Cessation. J Sci Dent. 2018 Dec;8(2):58–60. https://doi.org/10.5005/jsd-8-2-58
36. Budukh A, Shah S, Kulkarni S, Pimple S, Patil S, Chaukar D, et al. Tobacco and cancer awareness program among school children in rural areas of Ratnagiri district of Maharashtra state in India. Indian J Cancer. 2022 Jan-Mar;59(1):80-86. *DOI:* 10.4103/ijc.IJC_629_19
37. Singh A, Budukh AM, Chaturvedi P, Dikshit R. Systematic review on telephonic Quitline and its effects on smokeless tobacco. Int J Noncommunicable Dis. 2019 Sep;4(3):65.

*DOI:* 10.4103/jncd.jncd_26_19

357 38. Bhavya RCR, Musale D, Kadam D, Lanjekar K, Ogale G, Bhise M, et al. Challenges faced by
358 tobacco quit line services during the COVID-19 pandemic, Journal of Substance Use. Jan 2023 359	https://doi.org/10.1080/14659891.2023.2167744

360

361

Supporting Figure

[Click here to access/download](https://www.editorialmanager.com/pone/download.aspx?id=34340877&guid=f856e79b-68d7-428d-b24a-f4c7d4a9ce1a&scheme=1)

[Supporting Information S1 Fig.pdf](https://www.editorialmanager.com/pone/download.aspx?id=34340877&guid=f856e79b-68d7-428d-b24a-f4c7d4a9ce1a&scheme=1)

1. 1 in number of person likely to develop

   AAR of incidence per 100,000	cancer in lifetime
