# Supplementary material for: Burden of tobacco-related cancers in urban, semi-urban and rural setting of Nepal: Findings from population-based cancer registries 2019
Source: PLoS One. 2024 May 28;19(5):e0300271. doi: 10.1371/journal.pone.0300271 (PMC11132438; doi:10.1371/journal.pone.0300271)
Supplement: Attachment — Submitted filename: Response to Revierwers.docx [file pone.0300271.s003.docx]

Dear Editors,

Warm greetings from Nepal!

We thank you all for your generous review and insightful comments on the manuscript. The manuscript is now edited addressing all concerns of academic editor as well as reviewers. We have responded each query below. We believe that manuscript is now suitable for publication.

1. Though cigarette consumption is decreasing in Nepal, smokeless tobacco users are increasing in Nepal. How will your paper contribute to reducing such a burden? Explain introduction.

Response: We have included all cancers that are associated to be tobacco use (tobacco smoking and smokeless tobacco consumption). We found that urban and rural regions had higher risk for cancer to be associated with tobacco smoking and semi-urban region had high risk for cancers to be associated with smokeless tobacco use. Thus, This study certainly helps to prioritize tobacco control program in different regions of the country.

2. The method needs to be extended. It means providing in detail. The time period of Data collection is not clear.

Response: Methodology is now described in details including data collection period. Actually data collection in population-based cancer registry is ongoing and systematic process. We already registered used PBCR data of 1^st^ January 2019 to 31^st^ December 2019.

3. There is no consistency in writing. For Example, In the text, it is written male and female while in the table it is men and women.

Response: Corrected.

4. Please provide the number of male and female participants in the table so the reader can understand the % in the table. Also, provide footnotes for explaining %.

Response: Added and corrected

5. Authors have mentioned limitations but not strengths of the
      study. Please mention it.

Response: Limitation and strengths are mentioned.

6. The definition of Urban-rural and semi-urban areas is not clear.

Response: The regions were categorized on the basis of availability of medical facilities (diagnostics and treatment centers) Literacy rate, and transportation facilities. The details can be found in previous publication. reference no.14

7. The calculation of cumulative risk is not clear. Please provide Step-by-step methods.

Response: Calculation was done based on Day NE (1987).

8. 0-79 years. Can you clarify on this age group? How 0 years is related to TRC?

Response: It was not 0-79, It was 0-74. We were tried to provide baseline evidence on TRCs irrespective of age and other factors. Further studies can be done on this regards.

9. Why authors focused on place of residence but not age and other demographic factors? ( See tables)

Response: Due to inadequate documentation, PBCR couldn't able to collect data on all demographic variables and risk factors for all patient registered. We recommend further studies to identify association of TRCs and age, and other risk factors.

10. It is not clear how incidence rate were computed in this study?

Response: Incidence amd mortallty rate were computed by dividing all incidence and mortality cases of TRCs (diagnosed from 1^st^ January 2019 to 31^st^ December 2019 as well as dead in same year) respectively by same year population of the regions, and multiplied by 100,000. Which than further standardized using world standard population.

Thank you in advance for your consideration

Wish you very happy New Year 2024

Uma Kafle Dahal

On behalf of all authors
