# Supplementary material for: Burden of tobacco-related cancers in urban, semi-urban and rural setting of Nepal: Findings from population-based cancer registries 2019
Source: PLoS One. 2024 May 28;19(5):e0300271. doi: 10.1371/journal.pone.0300271 (PMC11132438; doi:10.1371/journal.pone.0300271)
Supplement: Attachment — Submitted filename: Response to Reviewers.docx [file pone.0300271.s004.docx]

Date 16^th^ February 2024

Dear editors and reviewers

The PLOS ONE,

Greetings from Nepal!

I am grateful for your time to review the manuscript and insightful comments which support us to make manuscript more meaningful. In response to your email requiring revision, I went through the manuscript as well as the list of reference. I have checked it again for its completeness and correctness to each article cited in our manuscript using Pub med and google scholars database as well as on the latest version of ZOTERO as well. I found none of the articles cited in our manuscript has been retracted. I would be grateful if you support us to identify any articles cited in our manuscript deemed to be confused.

I have changed source of information for line 33 (Reference no.2), and did some revision in the manuscript as suggested by reviewer which has been highlighted in tract change mode.

In response to the concern raised by Editor #4

First of all, I would like to thank you for your insightful revision on the manuscript. As the data collected by population-based cancer registry. It is always a census based where each and every cancer cases diagnosed in the specified period will be registered. Data was collected using structured questionnaires which includes variables recommended by International Association of cancer registries (IACR).

Concern about ethical consideration, secondary data was collected from health facilities taking prior permission from concerned authorities, and all the ethical consideration including individual consent taking was applied while collecting primary data through face-to-face method from patient or his/her family members. I have mentioned about the ethical consideration practices we followed during data collection.

Urban, semi-urban and rural regions was categories on the basis of geographical locations, and availability of facilities in the regions which was further clarified in our previous publication (reference no.14). I have done revision addressing the suggestion received from reviewer.

Thank you

Uma kafle Dahal

(on behalf of all authors)
